# The effect of marine n-3 polyunsaturated fatty acids on cardiac autonomic and hemodynamic function in patients with psoriatic arthritis: a randomised, double-blind, placebo-controlled trial

**DOI:** 10.1186/s12944-016-0382-5

**Published:** 2016-12-12

**Authors:** Salome Kristensen, Erik Berg Schmidt, Annette Schlemmer, Claus Rasmussen, Esther Lindgreen, Martin Berg Johansen, Jeppe Hagstrup Christensen

**Affiliations:** 1 Department of Rheumatology, Aalborg University Hospital, Reberbansgade 14, 9000 Aalborg, Denmark; 2 Department of Cardiology, Aalborg University Hospital, Department of Clinical Medicine, Aalborg University, 9000 Aalborg, Denmark; 3 Department of Rheumatology, North Denmark Regional Hospital, 9800 Hjørring, Denmark; 4 Department of Cardiology and Unit of Clinical Biostatistics and Bioinformatics, Aalborg University Hospital, 9000 Aalborg, Denmark; 5 Department of Nephrology, Aalborg University Hospital, Department of Clinical Medicine, Aalborg University, 9000 Aalborg, Denmark

**Keywords:** Psoriatic arthritis, n-3 PUFA, Heart rate variability, Cardiac autonomic function, Pulse wave velocity, Arterial stiffness

## Abstract

**Background:**

The aim of this study was to investigate the effect of marine n-3 polyunsaturated fatty acids (PUFA) on cardiac autonomic function and vascular function in patients with psoriatic arthritis.

**Methods:**

The study was conducted as a randomized, double-blind, placebo-controlled trial, where 145 patients with psoriatic arthritis were supplemented with 3 g of n-3 PUFA or olive oil (control) daily for 24 weeks. Blood pressure, heart rate, heart rate variability (HRV), central blood pressure, pulse wave velocity (PWV) and fatty acid composition of granulocytes, were determined at baseline and after supplementation.

**Results:**

At baseline we found a significant difference in the mean of all normal RR intervals (inverse of heart rate, vary from beat to beat) when comparing subjects with the highest vs the lowest fish intake (*p* = 0.03). After supplementation for 24 weeks there was a trend towards an increase in RR (*p* = 0.13) and decrease in heart rate (*p* = 0.12) comparing the n-3 PUFA group with the control group. However, per-protocol analysis showed significantly increased RR (*p* = 0.01) and lowered heart rate (*p* = 0.01) in the n-3 PUFA supplemented patients compared with controls. Blood pressure, PWV and Central blood pressure did not change after supplementation with n-3 PUFA. Adjustment for disease activity and conventional cardiovascular risk factors did not change the results.

**Conclusions:**

Marine n-3 PUFA increased RR intervals in patients with psoriatic arthritis which may suggest a protective effect of n-3 PUFA against cardiovascular disease in this population.

**Trial registration:**

Clinicaltrials.gov Identifier: NCT01818804

## Background

Psoriatic arthritis (PsA) is a chronic inflammatory joint disease occurring in 6–39% of patients with psoriasis and with a prevalence in the general population of approximately 0.2% [1–3].

Patients with inflammatory diseases such as rheumatoid arthritis and systemic lupus erythematosus are at an increased risk of cardiovascular disease (CVD) [4] and recent observational studies indicate that PsA patients also have an increased cardiovascular mortality and morbidity [5, 6]. Accelerated atherosclerosis due to inflammation, and autonomic dysfunction can both play a role in the pathogenesis of CVD in patients with PsA in addition to conventional risk factors for CVD such as smoking, hypertension, hypercholesterolemia and diabetes mellitus [7].

Several studies have indicated that chronic inflammation may impair autonomic cardiac regulation leading to a decrease in heart rate variability (HRV) [8, 9]. A low HRV has been identified as an independent predictor of coronary heart disease [10], as well as malignant ventricular arrhythmias and sudden cardiac death [11–15]. Also, arterial stiffness has been recognised as an independent predictor of CVD [16, 17]. Studies using non-invasive methods such as pulse wave velocity (PWV) for evaluation of CVD risk have revealed increased arterial stiffness in patients with PsA [18–20].

A beneficial effect of marine n-3 polyunsaturated fatty acids (PUFA) on CVD has been suggested from several epidemiological studies, experimental data and clinical trials [21]. Interestingly, the major marine n-3 PUFA, eicosapentaenoic acid (EPA; 20:5n-3) and docosahexaenoic acid (DHA; 22:6n-3), have been shown to have anti-inflammatory effects [22] and beneficial effects on blood pressure (BP) and cardiac autonomic function [23, 24].

The present study aimed to examine whether supplementation with a moderate to high (3 g) daily dose of marine n-3 PUFA for 24 weeks had a beneficial effect on cardiac autonomic and hemodynamic function represented by BP, heart rate (HR), HRV, PWV and central BP in patients with PsA.

## Methods

### Study design

The study was designed as a randomized, double-blind, placebo-controlled trial. The patients were randomly assigned in blocks of five by a computer-generated block sequence. For 24 weeks patients were assigned to daily intake of six capsules containing either 3 g of n-3 PUFA (50% EPA and 50% DHA) or 3 g of olive oil (approximately 80% of oleic acid and 20% linoleic acid). Investigators, patients and research staff were blinded to the supplementation codes. Patients were asked to maintain their usual diet during the entire study. The study was conducted in a 2 year period between 2013 and 2015. Clinical assessment, blood samples, HRV and PWV were carried out at baseline and after 24 weeks. The study was conducted in accordance with the Declaration of Helsinki and registered at ClinicalTrials.gov (NCT01818804). Furthermore the study was monitored by Good Clinical Practice (GCP) inspectors and the GCP ethical and scientific quality requirements were followed.

### Subjects

Patients with PsA defined by Classification criteria for psoriatic arthritis (CASPAR) [25] were enrolled from the Department of Rheumatology, Aalborg University Hospital and Department of Rheumatology, Vendsyssel Hospital, in Denmark. The inclusion criteria were PsA in adults above 18 years of age with any disease activity while exclusion criteria were documented known cardiac arrhythmias, treatment with biological drugs, or treatment with oral corticosteroids. Patients using fish oil supplements (*n* = 2) underwent a wash-out period of at least 8 weeks before inclusion.

Compliance was assessed by counting capsules during the last visit. Patients were defined as non-compliant if missing >15% of capsules and these patients were not included in the per-protocol analysis.

All participants gave their written informed consent and the regional ethics committee of Northern Region DK, approved the study (reference number N20120076).

### Clinical assessment

At baseline, duration of PsA, medical history, smoking habits and diets were obtained. Medical history of diabetes mellitus, hypertension and dyslipidemia was assessed and was defined as present if the patient received dietary or medical therapy for the condition. A food questionnaire was used to assess patients’ fish consumption at lunch and dinner. A score for fish intake was given according to the following: never eating fish = 1; eating fish once a month =2; eating fish two to three times a month = 3; eating fish once a week = 4; eating fish two to three times a week = 5; and eating fish at least once daily = 6.

At both visits conventional cardiovascular risk factors such as smoking habits, BP, body mass index (BMI) and waist to hip ratio (WHR) were assessed. Additionally, a clinical evaluation was performed, consisting of 68 tender joint count, 66 swollen joint count, disease activity score (DAS66/68) and psoriatic skin area involvement (PASI).

### Blood samples

Blood samples were taken non-fasting for assessment of fatty acid composition of granulocytes and routine laboratory evaluation including plasma levels of C-reactive protein (CRP). Granulocytes were isolated from whole blood and their fatty acid composition were determined by gas chromatography with a Chrompack CP-9002 gas chromatograph (Varian, Middelberg, The Netherlands) and expressed as weight percent (wt %) of total fatty acids. The following fatty acids were evaluated in the further analyses: eicosapentaenoic acid (EPA; 20:5n-3), docosahexaenoic acid (DHA; 22:6n-3), docosapentaenoic acid (DPA; 22:5n-3), arachidonic acid (AA; 20:4n-6), oleic acid (18:1n-9), linoleic acid (LA; 18:2n-6), palmitic acid (16:0) and stearic acid (18:0).

### HRV

Five min. HRV recordings were obtained with SphygmoCor Technology (SphygmoCor, Software version 8.2; AtCor Medical, Sydney, NSW, Australia) in each patient. HRV was recorded according to current recommendations [26] with measurements obtained in the morning hours after resting for 15 min in a room with a constant temperature of 20 °C. Patients were instructed not to smoke and avoid alcohol and caffeine-containing beverages within 12 h prior to investigation. A trained technician blinded to the type of supplement performed these analyses. The patients were in a supine position (resting) for 10 min, breathing spontaneously without talking. HRV were analysed in the time-domain and the following variables were obtained:

- HR: heart rate
- RR: mean of all normal RR intervals during the 5 min recording (inverse of heart rate, vary from beat to beat)
- SDNN: standard deviation of all normal RR intervals in the 5 min recording
- SDNNindex: mean of the standard deviation of all the normal RR intervals
- pNN50: percentage of successive RR-interval differences > 50 ms
- RMSSD: square root of the mean of the sum of the squares of differences between adjacent intervals

### PWV

PWV and pulse wave analysis were performed non-invasively with the Sphygmocor system (AtCor Medical, Sydney, NSW, Australia), as described previously [27] and according to international recommendations [28]. All measurements were made in duplicate by a single trained operator and the mean of the two values was used in the analysis. Carotid-radial and carotid-femoral PVW were measured using arterial tonometry. The measurements were obtained under the same conditions as described under HRV. The patients were in a supine position and all measurements were performed on the right side extremities. The surface distance was measured with a tape measure as a straight line from the suprasternal notch to the carotid location (proximal pulse) and subtracted the distance from the suprasternal notch to the radial or femoral location (distal pulse) [29, 30]. The pressure wave transit time was determined as the time between the R-wave of the ECG and the proximal pulse subtracted from the time between the R-wave of the ECG and the distal pulse. PWV was subsequently calculated by dividing the surface distance by the pressure wave transit time.

The central BP was estimated using the SphygmoCor® device. After 10 min of rest in the supine position, brachial BP was measured three times at 2-min intervals on the left arm with an automatic Microlife® device, and the last measurement was taken as representative of brachial artery BP. Hereafter, radial artery pressure waveforms of the right arm were sampled. Using the validated generalized transfer function, central BP was estimated using brachial systolic and diastolic BP [31, 32]. Aortic augmentation index (AIx) was standardized to a HR of 75 beat per minute to minimize the effect of HR.

### Statistical Analysis

All statistical analyses were performed using Stata: Release 13 (StataCorp LP, TX, US). HRV measurements were primary outcome and PWV measurements secondary outcome.

We hypothesized that intervention with n-3 PUFA would increase RR. Based on previous literature [33, 34] and to achieve α = 0.05 and 1-β = 0.80 we needed a sample size with 63 subjects in each group.

The score for fish intake at baseline was grouped according to tertiles. The content of DHA and EPA in granulocytes were also grouped according to tertiles.

The difference in the continuous outcomes between baseline and 24 weeks after randomization was compared between the two treatment groups in a one-way analysis of variance (ANOVA). Equality of variances between the treatment groups was assessed using Bartlett’s test. Due to the potential for confounding after the random treatment assignment an analysis of covariance (ANCOVA) was performed. Prior to this analysis a check for collinearity between the covariates was performed and the model was modified accordingly. In this model equality of variances was assessed using Levene’s test. All analyses were performed both as intention to treat and per-protocol (patients who completed the entire clinical trial according to the protocol and consumed > 85% of the assigned supplement) analyses. The ANCOVA analyses were controlled for age, sex, smoking status, presence or absence of diabetes mellitus, hypertension and hypercholesterolemia, use of nonsteroidal anti-inflammatory drugs (NSAID) and DAS66/68.

Differences were considered significant with a *p*-value of <0.05 (two-tailed).

## Results

**Fig. 1:**
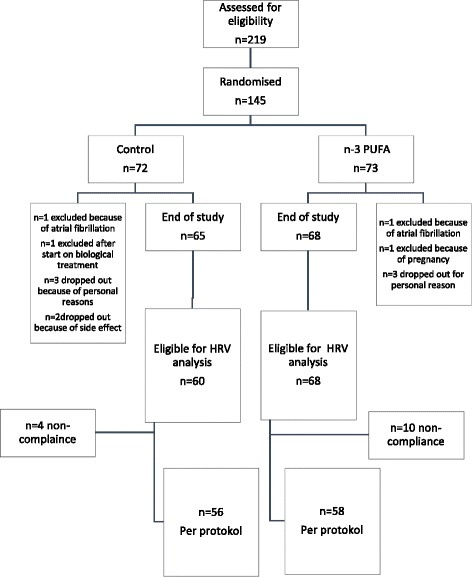
Flow diagram of the study participants

A total of 145 patients were enrolled and 133 patients (92%) completed the study. Seven patients in the control arm and five patients in the n-3 PUFA arm withdrew from the study before last visit and five patients were excluded because of insufficient HRV recordings at baseline. Ten patients in the n-3 PUFA group and four in the control group were defined as noncompliant according to the number of capsules returned and were not included in the per-protocol analysis. Figure 1 shows the study flow diagram.

The n-3 PUFA and the control groups were comparable regarding baseline characteristics, apart from number of current smokers and number of patients with known hypercholesterolemia (Table 1). Thus, adjustments were made for these to parameters in the final analysis. Patients excluded from final analysis did not differ from patients eligible for final analysis in regard to baseline characteristics (data not shown). Nine participants in the n-3 PUFA supplemented group and six participants in the control group reported mild gastrointestinal adverse effect. In 13 of these participants the side effects was avoided by changing the intake of capsules from six capsules once daily to two capsules three times daily. The remaining two participants were in the placebo group and dropped out because of nausea and mild diarrhea.

**Table 1:** Baseline demographics, biochemical and hemodynamic characteristics of the 143 patients with psoriatic arthritis

		n-3 PUFA (*N* = 72)	Control (*N* = 71)	Total (*N* = 143)
Covariates	Age			
	Mean age	53.2 (11.4)	50.7 (11.5)	52.0 (11.5)
	Sex			
	- Female	40 (55.6)	43 (60.6)	83 (58.0)
	- Male	32 (44.4)	28 (39.4)	60 (42.0)
	Smoking			
	- Non-smoker	36 (50.0)	41 (57.7)	77 (53.8)
	- Former smoker	27 (37.5)	14 (19.7)	41 (28.7)
	- Current smoker	9 (12.5)	16 (22.5)	25 (17.5)
	Documented coronary heart disease	4 (5.6)	4 (5.6)	8 (5.6)
	Hypertension	21 (29.2)	20 (28.2)	41 (28.7)
	Hypercholesterolemia	18 (25.0)	11 (15.5)	29 (20.3)
	NSAID use	40 (55.6)	32 (45.1)	72 (50.3)
Outcomes	Systolic BP, mmHg	138 (18)	134 (19)	136 (19)
	Diastolic BP, mmHg	83 (11)	82 (12)	82 (12)
	Hip and waste ratio	1.1 (0.1)	1.2 (0.1)	1.1 (0.1)
	Body Mass Index	28.6 (5.7)	28.0 (5.0)	28.3 (5.4)
	Disease Activity Score	2.5 (0.9)	2.7 (0.9)	2.6 (0.9)
	CRP mg/l	4.6 (4.2)	6.1 (7.7)	5.3 (6.2)
	PASI	2.2 (3.0)	2.3 (4.0)	2.3 (3.5)
	Total cholesterol, mmol/l	5.0 (1.0)	4.8 (0.8)	4.9 (0.9)

Data are given as mean (sd) or *n* (%) as appropriate

*N* non-missing values, *BP* Blood pressure

### Baseline analyses

**Fig. 2:**
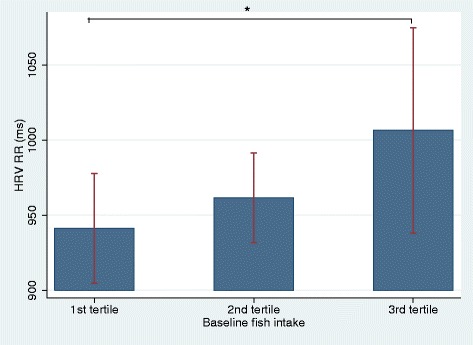
The relation between baseline RR (ms) and fish intake presented in tertiles. Footnote: RR: Mean of all normal RR-intervals in HRV recording; *: Significant difference in RR between the lower and the upper tertile

**Fig. 3:**
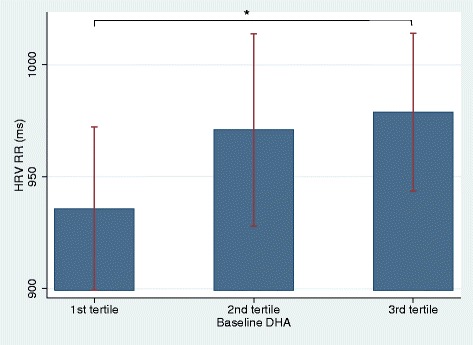
The relation between baseline RR (ms) and content of DHA in granulocytes presented in tertiles. Footnote: HRV: Heart Rate Variability; RR: Mean of all normal RR-intervals in HRV recording; DHA: docosahexaenoic acid; *: Significant difference in RR between the lower and the upper tertile

Patients with the highest fish intake had a significantly higher RR than patients with the lowest intake (*p* = 0.03). Also, patients in the tertile with the highest content of DHA in granulocytes had the highest RR (*p* = 0.04) whereas the content of EPA in granulocytes was not associated with RR. The associations between dietary fish intake and RR seemed to be dose dependent (Figs. 2 and 3). There were no association between DAS66/68 and RR at baseline.

### Intervention analyses

The group randomized to n-3 PUFA had a significant increase in total content of n-3 PUFA (DHA + EPA + DPA), DHA and EPA in granulocytes from baseline to study end compared to the control group. There were also a significant decrease in the granulocyte content of AA and LA in the n-3 PUFA supplemented group compared to control group (Table 2).

**Table 2:** Content of fatty acids in granulocytes presented as means with 95% confidence intervals at baseline and after 24 weeks of supplementation for both groups

	n-3 PUFA (*n* = 68)			Control (*n* = 65)			
	Baseline	Week 24	Difference (CI)	Baseline	Week 24	Difference (CI)	P
Palmitic acid (16:0) wt.%	12.32	12.40	0.08 (−0.04 - 0.21)	12.38	12.37	−0.00 (−0.12 - 0.12)	0.33
Stearic acid (18:0) wt %	17.36	17.35	−0.01 (−0.12 - 0.11)	17.41	17.30	−0.11 (−0.24 - 0.01)	0.20
Oleic acid (18:1n-9) wt.%	32.90	33.21	0.30 (0.03; 0.57)	32.97	33.44	0.47 (0.24; 0.70)	0.35
Linoleic acid (18:2n-6) wt. %	9.12	8.62	−0.50 (−0.80; −0.21)	9.32	9.21	−0.11 (−0.26; 0.05)	0.02
AA (20:4n-6) wt %	13.05	11.44	−1.61 (−1.84; −1.38)	13.01	12.80	−0.20 (−0.37; −0.04)	<0.01
Total n-6 wt %	22.17	20.05	−2.12 (−2.47 - -1.76)	22.32	22.01	−0.31 (−0.51 - -0.11)	<0.01
EPA wt %	0.60	1.99	1.40 (1.24; 1.56)	0.53	0.50	−0.03 (−0.08; 0.02)	<0.01
DPA wt %	1.58	2.61	1.03 (0.86; 1.21)	1.46	1.42	−0.04 (−0.10; 0.02)	<0.01
DHA wt %	1.21	2.10	0.89 (0.77; 1.00)	1.14	1.11	−0.02 (−0.07; 0.02)	<0.01
Total n-3 PUFA (DHA + EPA + DPA) wt %	3.39	6.71	3.32 (2.92; 3.71)	3.13	3.03	−0.10 (−0.22; 0.03)	<0.01

*AA* arachidonic acid, *DHA* docosahexaenoic acid, *EPA* eicosapentaenoic acid, *DPA* docosapentaenoic acid, *wt. %* weight percent, *P* P for the difference between the two groups of supplement

#### Intention to treat analysis

After supplementation for 24 weeks there was a trend towards an increase in RR (*p* = 0.06) and a decrease in HR (*p* = 0.12) comparing the n-3 PUFA group with the control group (Table 3).

**Table 3:** Intention to treat data with no adjustments

	n-3 PUFA (*n* = 68)			Control (*n* = 60)			
	Baseline	Week 24	Difference (CI)	Baseline	Week 24	Difference (CI)	P
Heart rate, min^−1^	63.83	63.29	−0.61 (−1.92; 0.70)	63.39	64.38	0.96 (−0.55; 2.47)	0.12
RR, ms	956.55	969.94	13.38 (−5.06; 31.83)	964.02	950.53	−13.48 (−35; − 8.69)	0.06
PNN50 %	10.68	11.29	0.62 (−3.25; 4.48)	15.83	14.37	−1.46 (−4.76; 1.84)	0.42
SDNN ms	49.37	48.12	−1.24 (−8.46; 5.97)	49.71	47.41	−2.30 (−6.79; 2.20)	0.81
RMSSD ms	36.59	37.58	0.99 (−7.13; 9.12)	39.84	39.15	−0.69 (−6.23; 4.85)	0.73
Peripheral systolic BP mmHg	138.20	134.53	−3.67 (−6.69; −0.65)	134.41	133.18	−1.23 (−4.59; 2.14)	0.28
Peripheral diastolic BP mmHg	82.61	81.82	−0.79 (−2.35; 0.77)	82.36	80.92	−1.44 (−3.26; 0.39)	0.59
PWV m/s	7.80	7.81	0.01 (−0.44; 0.46)	7.40	7.48	0.08 (−0.33; 0.49)	0.82
Central systolic BP mmHg	114.82	112.24	−2.58 (−4.84; −0.32)	113.29	111.38	−1.91 (−4.77; 0.95)	0.71
Central diastolic BP mmHg	96.04	93.76	−2.28 (−4.10; −0.47)	95.45	94.17	−1.29 (−3.51; 0.94)	0.49
PWA AIx	26.42	27.54	1.12 (−0.56; 2.80)	26.97	25.82	−1.15 (−2.78; 0.47)	0.05

Outcomes presented as means with 95% confidence intervals at baseline and after 24 weeks of supplement for both groups

*CI* Confidence Interval, *P* P for difference between the two groups of supplement, *HRV* Heart rate variability, *PWV* Pulse wave velocity, *BP* blood pressure, *AIx* central Augmentation Index

There were no significant change in BP, PWV or central BP in the n-3 PUFA supplemented group or between the n-3 PUFA and control group. Adjustment for conventional CVD risk factors and DAS66/68 did not affect the results.

DAS66/68 and CRP were not associated with HRV or PWV.

#### Per-protocol analysis

Analyses of outcomes after 24 weeks revealed a significant increase in RR and decrease in HR in the fish oil supplemented group and there was a significant difference in changes in RR (*p* = 0.03) and HR (0.02) between the n-3 PUFA and control group (Table 4). In contrast, there were no significant change in BP, PWV or central BPs in the n-3 PUFA supplemented group or between the n-3 PUFA and control group. Adjustment for conventional CVD risk factors and DAS66/68 did not change the results (Table 5).

**Table 4:** Per-protocol data with no adjustments

	n-3 PUFA (*n* = 58)			Control (*n* = 56)			
	Baseline	Week 24	Difference (CI)	Baseline	Week 24	Difference (CI)	P
Heart rate	63.24	61.73	−1.51 (−2.89; −0.13)	63.39	64.38	0.98 (−0.54; 2.50)	0.02
RR ms	964.14	990.39	26.25 (6.21; 46.30)	964.02	950.53	−13.48 (−35.66; 8.69)	0.01
PNN50 %	11.39	11.65	0.26 (−4.06; 4.58)	15.83	14.37	−1.46 (−4.76; 1.84)	0.52
SDNN ms	51.56	49.36	−2.20 (−9.96; 5.56)	49.71	47.41	−2.30 (−6.79; 2.20)	0.98
RMSSD ms	39.10	37.85	−1.25 (−9.41; 6.91)	39.84	39.15	−0.69 (−6.23; 4.85)	0.91
Peripheral BP Systolic mmHg	137.67	134.29	−3.38 (−7.03; 0.26)	134.41	133.18	−1.23 (−4.59; 2.14)	0.39
Peripheral BP Diastolic mmHg	81.96	80.88	−1.08 (−2.89; 0.74)	82.36	80.92	−1.44 (−3.26; 0.39)	0.78
PWV m/s	7.66	7.61	−0.04 (−0.51;- 0.43)	7.40	7.48	0.08 (−0.33; 0.49)	0.70
Central BP systolic mmHg	114.62	112.17	−2.44 (−5.17; 0.28)	113.29	111.38	−1.91 (−4.77; 0.95)	0.79
Central BP diastolic mmHg	95.50	93.13	−2.37 (−4.49; −0.24)	95.45	94.17	−1.29 (−3.51; 0.94)	0.49
PWA AIx	26.42	27.51	1.12 (−0.56; 2.80)	27.02	25.84	−1.15 (−2.78; 0.47)	0.06

Outcomes presented as means with 95% confidence intervals at baseline and after 24 weeks of supplement for both groups

*CI* Confidence Interval, *P* P for difference between the two groups of supplement, *PWV* Pulse wave velocity, *BP* blood pressure, *AIx* central Augmentation Index

**Table 5:** Per-protocol analysis

	Estimated effect (95% CI)	F statistic	P	N
RR ms	35.26 (3.00 to 67.53)	4.70	0.03	114
PNN50 %	1.81 (−3.99 to 7.61)	0.38	0.54	114
SDNN ms	−0.05 (−9.49 to 9.39)	0.00	0.99	114
RMSSD ms	1.35 (−9.06 to 11.76)	0.07	0.80	114
Peripheral systolic BP, mmHg	−1.48 (−6.25 to 3.29)	0.38	0.54	117
Peripheral diastolic BP, mmHg	0.26 (−2.45 to 2.96)	0.03	0.85	117
Pulse Wave Velocity, m/s	−0.15 (−0.83 to 0.54)	0.18	0.67	111
Central systolic BP mmHg	−0.90 (−5.23 to 3.44)	0.17	0.68	117
Central diastolic BP mmHg	−1.57 (−4.94 to 1.79)	0.86	0.36	117
PWA AIx	2.91 (0.12 to 5.71)	4.26	0.04	117

ANCOVA analysis of differences in outcomes from baseline to 24 weeks comparing n-3 PUFA to control. Analysis are controlled for age, sex, smoking, diabetes mellitus, hypertension, blood pressure, hypercholesterolemia, NSAID treatment and Disease activity scores. N are numbers of patients

*CI* Confidence Interval, *HRV* Heart rate variability, *BP* Blood pressure, *PWV* Pulse wave velocity, *AIx* central Augmentation Index

## Discussion

With 145 participants and only 8% not completing, this study is the largest investigation of the effect of marine n-3 PUFA on cardiovascular function in patients with PsA. We found that supplementation with 3 g n-3 PUFA daily for 24 weeks had a beneficial effect on autonomic control of the heart in PsA patients by decreasing heart rate and increasing RR interval.

At baseline we found a significantly higher RR in the patients with the highest fish intake. Baseline analysis also showed that the content of DHA in granulocytes was positively associated with RR. These associations seemed to be dose dependent and the results suggest beneficial effect of dietary fish consumption on the cardiac autonomic tone in patients with PsA.

After intervention for 24 weeks there was a significant shift in the PUFA composition in the active group with an increase in the granulocyte content of n-3 PUFA and a decrease in content of AA and LA. In the intention to treat analysis there was a trend towards increased RR and thereby a reciprocal lowering of HR. However, the per-protocol analysis revealed a significant increase in RR and decrease in HR in the n-3 PUFA supplemented group.

HRV is considered a useful and reliable measurement of cardiac autonomic tone [35]. A depressed HRV indicates an increased cardiovascular risk in the general population [11] and in patients with known CVD [36]. Evidence also suggests that HRV improves with fish consumption and intervention with n-3 PUFA [37]*.* The positive association between fish intake and RR at baseline and the increase in RR after intervention with n-3 PUFA found in this study of patients with PsA is in line with previous studies of other high-risk patients and healthy subjects [38]. The effect of n-3 PUFA on HR in our study is also consistent with previous data showing that n-3 PUFA reduces resting HR [39, 40], an important risk marker for cardiovascular disease [41]*.* As a surrogate marker for cardiac autonomic tone, HRV can indicate changes mediated by n-3 PUFA at the level of cardiac efferent stimuli [42, 43]. Interestingly, in our study baseline content of DHA but not EPA in granulocytes was positively associated with RR, which is consistent with findings of previous studies [44, 45]. DHA is most abundant in the heart, brain and nervous system membrane lipids [46] and therefore might be more influential regarding effects on cardiac autonomic function.

Interactions between the autonomic nervous system and the immune system have been reviewed recently and direct autonomic innervation and non-synaptic communication with lymphoid organs has been shown [47]. Abnormalities in the autonomic nervous system as well as cardiovascular autonomic dysfunction has been reported in patients with inflammatory rheumatic diseases [48]. A few studies have investigated HRV in PsA and demonstrated attenuated HRV. In a study with 38 patients with PsA and 25 healthy controls using 5-min HRV Gaydukova et al. [49] found that SDNN was 65.1 ms and pNN50 12.9% in the PsA patients whereas in healthy controls SDNN and pNN50 were higher (83.2 ms and 20.6%, respectively). Proietti et al. [50] also used short-term HRV in 26 patients with psoriasis and 27 healthy controls and found a significant difference in RMSDD between patients with psoriasis (39.4 ms) and the controls (53.2 ms). Our results revealed a baseline SDNN of 50 ms, pNN50 of 13%, and RMSSD of 38 ms, supporting an attenuation of HRV in patients with PsA. A likely explanation for a low HRV in PsA is the presence of systemic inflammation leading to a decreased parasympathetic regulation of cardiac autonomic tone [51, 52]. In this study, DAS66/68 and CRP were not associated with HRV or PWV. However, DAS and CRP may be a more important marker of disease activity in patients with RA [53] than in patients with PsA [47, 54, 55].

In a small study with 20 patients with PsA, Syngle et al. observed improvement in autonomic dysfunction after treatment with different synthetic DMARDs over 12 weeks [33]. However, no treatment strategy for cardiac autonomic dysfunction in PsA has yet been demonstrated. Thus, the possible beneficial effect of n-3 PUFA found in our study may be of importance in the approach towards an improved cardiac autonomic function in PsA.

n-3 PUFA is known to have a mildly antihypertensive effect [56]. Furthermore, studies and a meta-analysis of randomized and controlled human clinical trials examining the effect of n-3 PUFA on arterial stiffness has shown a reduction in arterial stiffness after treatment with less than 4 g/d of n-3 PUFA in populations with hypertension, diabetes mellitus, dyslipidemia, metabolic syndrome and obesity [57, 58].

In our study we were unable to demonstrate any changes in BP, PWV, central BP and AIx measurements. However, in most of the previous studies arterial stiffness was not assessed with carotid-femoral PWV (regarded as “golden standard”) as in our study. Supplementation with n-3-PUFA for 24 weeks might be too short a period to observe changes and modulation in the vessels measured by PWV. Patients in this study had a low mean disease activity score of 2.6 (sd = 0.9) at baseline and 75% of the patients received disease-modifying antirheumatic drugs. Thus, we investigated a patient group in remission and results may not apply to patients with a more severely disease activity. In other chronic inflammatory diseases, such as RA and systemic vasculitis, PWV and AIx were increased compared to controls, but only in patients with active disease [59, 60].

### Limitation of the study

Five minutes HRV was used to assess the function of the autonomic nervous system. This method limits the measurement of vagal predominance during nighttime. However, other studies assessing patients with PsA have also obtained 5 min HRV with results comparable to our findings.

The difference in HRV in the intention to treat and per-protocol results might be explained by non-compliance. Compliance and adherence to the study protocol might have been improved with administration of more concentrated capsules of n-3 PUFA resulting in fewer capsules per day for the patients.

Olive oil has often been used as control oil in studies investigating the effects of fish oil in patients with arthritis but olive oil itself may have some effect on inflammation [61]. However, marine n-3 PUFA have been shown to be superior to olive oil in several studies of CVD [37] and olive oil had no anti-arrhythmic effect in previous studies [62, 63]. The mean intake of monounsaturated fat in Denmark is 36 g/d [64] and therefore, adding 3 g/d of olive oil would not be expected to have a substantial effect on the results. Also, we found no significant changes in the content in granulocytes of oleic acid and linoleic acid (main components of olive oil) in the control group after 24 weeks of supplementation (Table 2).

## Conclusion

In conclusion, our study demonstrated a beneficial effect of n-3 PUFA on RR and HR in a well-characterized group of patients with PsA. The results may indicate a beneficial effect of n-3 PUFA on cardiac autonomic tone in patients with PsA and further large-scale studies are needed to demonstrate whether this translates into a reduction of CVD in these patients.

## Abbreviations

AA: Arachidonic acid
AI: Augmentation index
AIx: Aortic augmentation index
ANCOVA: Analysis of covariance
ANOVA: One-way analysis of variance
BMI: Body mass index
BP: Blood pressure
CASPAR: Classification criteria for psoriatic arthritis
CI: Confidence intervals
CRP: C-reactive protein
CVD: Cardiovascular disease
DAS: Disease activity score
DHA: Docosahexaenoic acid
DMARD: Disease-modifying antirheumatic drugs
DPA: Docosapentaenoic acid
EPA: Eicosapentaenoic acid
GCP: Good clinical practice
HR: Heart rate
HRV: Heart rate variability
LA: Linoleic acid
n-3 PUFA: n-3 polyunsaturated fatty acids
NSAID: Nonsteroidal anti-inflammatory drugs
PASI: Psoriatic skin area involvement
pNN50: Percentage of successive RR-interval differences > 50 ms
PsA: Psoriatic arthritis
PWV: Pulse wave velocity
RMSSD: Square root of the mean of the sum of the squares of differences between adjacent intervals
RR: Mean of all normal RR intervals during the 5 min recording
SD: Standard deviation
SDNN: Standard deviation of all normal RR intervals in the 5 min recording
SDNNindex: Mean of the standard deviation of all the normal RR intervals
